# Predictive Modeling for Inactivation of *Escherichia coli* Biofilm with Combined Treatment of Thermosonication and Organic Acid on Polystyrene Surface

**DOI:** 10.3390/foods13244002

**Published:** 2024-12-11

**Authors:** Emel Unal Turhan, Esabil Emrah Koca

**Affiliations:** Department of Food Technology, Faculty of Kadirli Applied Sciences, Osmaniye Korkut Ata University, 80760 Kadirli, Osmaniye, Turkey; dytesabil@gmail.com

**Keywords:** biofilm, *Escherichia coli*, termosonication, organic acid, multiple linear regression

## Abstract

The present study aimed to evaluate the antibiofilm effect of combined sonication treatment with organic acids on polystyrene surfaces and to develop a predictive model for the inactivation of *Escherichia coli* biofilms. Polystyrene plates containing *E. coli* biofilms were subjected to sonication using different inactivation solutions (PBS, lactic acid, and acetic acid) at varying temperatures (20 °C, 40 °C, and 50 °C) and durations (2 and 5 min). The effects of temperature, treatment duration, and inactivation solution on *E. coli* biofilm removal were statistically significant (*p* < 0.05). The use of organic acids, along with increased treatment time and temperature, led to a significant reduction in viable cell counts (0.43–6.21 log CFU/mL) and optical density (0.13–0.72 at OD600) of *E. coli* biofilms (*p* < 0.05). The highest *E. coli* biofilm inactivation, with a reduction of 6.21 CFU/mL and 0.72 OD, was achieved by combining organic acid and thermosonication at 50 °C for 5 min. A significant positive correlation was observed between test methods based on viable cell count and optical density (OD) measurements. According to multiple linear regression analysis results, the *R*^2^ values of the predictive models for biofilm inactivation, based on viable cell count and OD measurements, were 0.84 and 0.80, respectively. Due to its higher accuracy, the predictive model developed using viable cell count data is recommended for applications in the food industry and processing sectors.

## 1. Introduction

Biofilms are aggregates of microbial cells embedded within extracellular polymeric substances. Foodborne pathogens can form biofilms on food contact surfaces depending on temperature, pH, nutrient content, salinity, contact surface properties, and microorganism strain [1]. *Escherichia coli* O157:H7 biofilm is an especially severe threat to food safety and public health since the infectious dose of *E. coli* O157:H7 is low (<100 cells). *E. coli* can colonize food processing surfaces, including polystyrene, polyethylene, rubber, polycarbonate, polyurethane, glass, granite, marble, stainless steel, wood, and siltstones. Polystyrene and stainless steel are the most widely used materials in the food industry. Polystyrene microtiter plate assay is well suited for screening biofilm formation by numerous strains. It can provide relevant information on biofilm-forming abilities on other food contact surfaces [1,2,3,4,5]. The adhesion ability of *Escherichia coli* varies depending on the type of food contact surface. For instance, *E. coli* exhibits stronger attachment on wooden surfaces, reaching approximately 10 log CFU/cm^2^, compared to other surface types. In contrast, its adhesion to plastic, glass, and stainless steel surfaces is generally comparable, at approximately 8.5 log CFU/cm^2^. According to Park and Chen [6], the trend of *E. coli* biofilm formation on stainless steel surfaces (OD600nm = max. 1.49) was similar to that on polystyrene surfaces (OD600nm = max. 1.62). Cross-contamination of food by food-contact surfaces harboring low levels of pathogens has the potential to cause outbreaks [1]. Biofilms formed by foodborne pathogens are responsible for most outbreaks globally, making their presence in the food industry a significant concern and a serious threat to food safety. The formation of biofilms on food-contact surfaces by foodborne pathogens increases the likelihood of cross-contamination. Therefore, cleaning and sanitizing these surfaces are critical measures to prevent cross-contamination and mitigate the spread of foodborne illnesses. Various techniques have been employed to disinfect food contact surfaces contaminated with *E. coli*, including gaseous ozone, UV-C light, atmospheric plasma jets, natural extracts, and non-thermal dielectric barrier discharge (DBD) plasma. Among these methods, innovative approaches such as oregano (*Origanum vulgare* L.) essential oil vapors and non-thermal DBD plasma demonstrate significant potential for the decontamination of plastic surfaces [7,8]. However, removing biofilms presents a significant challenge, as biofilms formed by pathogens exhibit a better resistance to environmental stresses than their planktonic cells. The extracellular polymeric substance (EPS) matrix of biofilms provides structural support and protection to microbial communities, enabling their survival in harsh environmental conditions [2].

Current strategies for controlling pathogen biofilms primarily rely on physical methods, such as heating, ultrasonication, UV-V irradiation, cold oxygen plasma, and chemical approaches, including chlorine, hydrogen peroxide, ethanol, and organic acids [9]. In recent years, there has been an increasing emphasis on developing environmentally friendly and safe treatments for biofilm inactivation [10]. Among emerging technologies, ultrasound has gained attention as a promising method for effectively detaching biofilms from contact surfaces while simultaneously exerting bactericidal effects [11]. In particular, an effective biofilm reduction is provided with the combined treatment of ultrasound and organic acids on fresh produce. Limited studies are concerned with combination treatment of acid and ultrasound to decontaminate food contact surfaces. For example, Shao et al. [12] reported that the single acidic electrolyzed water treatment, single ultrasound treatment, and their combination caused 3.0, 0.8, and 4.8 log CFU/cm^2^ pathogen (*Salmonella* spp. and *Staphylococcus aureus*) biofilm reduction on stainless steel surfaces, respectively. In previous studies, single ultrasound or single organic acid treatment was mainly applied for the decontamination of food contact surface. Single treatment including ultrasound or organic acid exhibited antibiofilm activity depending on sonication temperature, sonication time, concentration, and type of organic acids [2,10,13,14,15,16,17]. The most commonly used organic acids in the food industry are acetic acid, lactic acid, citric acid, malic acid, and peracetic acid. The antimicrobial activity of these acids primarily depends on their undissociated form and pH levels [18]. When combined with organic acids for biofilm removal, ultrasound enhances the permeability of chemical disinfectants into biofilms by exposing inner cells through mechanical oscillation. This process facilitates chemical sanitizers’ more effective bactericidal action [2]. In biofilm inactivation, a combination of physical and chemical approaches can yield a more substantial decontamination effect [18,19]. When ultrasound is applied alone, its inactivation effect on various pathogens ranges from 0.5 to 1.98 log CFU/g. However, this effectiveness significantly increases when ultrasound is combined with other disinfection methods, such as organic acids, alcohols, essential oils, bacteriocins, and UV treatments. For example, combination treatment of US and 1% acetic acid or lactic acid caused approximately additional 1 log reduction in *E. coli* according to single treatment [20].

Ultrasonic processes have been usually performed at low temperatures. However, in recent years, thermosonication has been applied with a more significant effect on the inactivation of microorganisms than heat alone. For instance, D values with heat, ultrasound, and thermosonication were 0.79, 1.01, and 0.44 at 61 °C for *E. coli* K12, respectively [21]. Generally, thermosonication combines a mild heat of 37 to 75 °C with low-frequency ultrasound waves (20 kHz) treatment [22]. Thermosonication applications in the food industry offer numerous advantages, including improved product quality, reduced processing times, environmental sustainability, and lower associated hazards. The microbial inactivation mechanism of thermosonication is attributed to physical effects (such as cavitation and heat) and chemical effects (such as the formation of free radicals) [21,23]. Single microbial control strategies are often insufficient to eliminate pathogenic bacteria without causing damage to food contact or equipment surfaces. However, combining thermosonication with chemical agents enhances sanitation effectiveness while preventing damage to food contact surfaces and equipment from excessively intense treatment conditions, such as prolonged processing times, higher processing temperatures, and increased amounts of antimicrobial agents. During thermosonication, food contact surfaces are exposed to shorter treatment durations and lower disinfectant concentrations [22]. The effects of treatment conditions on microbial inactivation are often analyzed using predictive modeling. Various predictive models have been proposed to describe the elimination of foodborne pathogens. However, these models are case-dependent and influenced by environmental factors, bacterial strain variations, and processing parameters [24]. Using mathematical equations to predict microbial inactivation is critical in hazard analysis, establishing critical control points, and enhancing food safety. Notably, only a limited number of studies have addressed the predictive modeling of biofilm inactivation during sonication treatment. Linear and non-linear kinetic models, such as log-linear, log-linear shoulder, biphasic linear, logistic, multi-target, and single-target models, have been commonly employed to study the inactivation kinetics of microorganisms in various research efforts [2,24,25,26]. Esua et al. [24] reported that the inactivation of *Escherichia coli* on grass carp, achieved through the combined application of ultrasound and plasma-functionalized buffer, was effectively modeled using non-linear approaches, demonstrating high adjusted R^2^ values (0.962–0.999). Similarly, Zhao et al. [2] demonstrated that the inactivation kinetics of *E. coli* biofilms were well simulated using the modified Weibull model, achieving R^2^ values ranging from 0.81 to 0.97 and RMSE values between 0.04 and 0.71 to assess the effects of electrolyzed water combined with ultrasound. Furthermore, in another study, the response surface model (RS model) exhibited a strong fit, with an R^2^ value of 0.942 and an adjusted R^2^ of 0.916, for modeling the inactivation of *Shigella flexneri* using a combination of ultrasound, pH, and nisin [26]. On the other hand, the food industry requires more predictive models for effective sanitation procedures [14].

As an emerging technology, thermosonication and its combined treatments have been utilized in food products. However, the available data on the application of thermosonication for the inactivation of foodborne pathogens remain limited [22]. To our knowledge, there are no previous studies on biofilm inactivation with thermosonication on the food contact surface and its predictive modeling. The present study aimed to determine the efficacy of combined treatments with thermosonication and organic acids on removing *E. coli* biofilm on polystyrene surfaces and to predict this efficacy employing a multiple linear regression model.

## 2. Materials and Methods

### 2.1. Bacterial Strain, Culture Preparation and Organic Acids

*Escherichia coli* (NCTC 12241) was obtained from the culture collection of Osmaniye Korkut Ata University (Osmaniye, Turkey) and stored in Tryptic Soy Broth (TSB; Difco, Becton Dickinson, Sparks, MD, USA) supplemented with 20% glycerol (Sigma-Aldrich, Darmstadt, Germany) at −20 °C. The bacterial stock was initially streaked onto Tryptic Soy Agar (TSA; Difco) plates and incubated at 37 °C for 24 h. Subsequently, a single colony was inoculated into TSB and incubated overnight at 37 °C. The overnight culture, containing a consistent bacterial concentration of approximately 8 log CFU/mL, was used for all experiments. The final concentration of bacterial cells was standardized by measuring the optical density at 600 nm (OD600) using the ELISA Microplate Reader (Rayto-RT 6000 ELISA Microplate Reader, Range Rayto Life and Analytical Sciences Co., Ltd., Shenzhen, China), with a final OD600 value of 1.5 [1,3,15].

All inactivation treatment solutions were prepared immediately before each experiment and used within 30 min. The treatment solutions consisted of phosphate-buffered saline (PBS) as a control, 2% lactic acid (*v*/*v*; Sigma-Aldrich, Vienna, Austria), and 2% acetic acid (*v*/*v*; prepared from glacial acetic acid; Merck, Darmstadt, Germany). All solutions were prepared using demineralized water and maintained at room temperature (approximately 20 °C).

### 2.2. Biofilm Formation

Biofilm formation was performed according to the microplate method previously described by Yu et al. [11], Wang et al. [17], Castillo et al. [27], Amrutha et al. [28], and Bang et al. [29] with minor modifications. Accordingly, 3 mL of TSB with 1% overnight culture of *E. coli* strain was added to each well of a 12-well microplate (SPL Life Sciences, Greenpia Technology, Gyeonggi, Republics of Korea) and left incubation for 48 h at 37 °C. The medium containing planktonic cells was removed, and each well was washed three times with 3.5 mL of phosphate-buffered saline (PBS). After the washing procedures, the remaining part at the bottom of the wells was evaluated as a biofilm. The amount of this biofilm was evaluated with two different methods: optical density measurement and viable cell enumeration (Section 2.3 and Section 2.4). Biofilms obtained in this Section 2.2 were also used in the following experiments (Section 2.3, Section 2.4 and Section 2.5).

### 2.3. Crystal Violet Assays of Biofilms and Optical Density

*E. coli* biofilms in wells were stained with 3.5 mL of 0.1% crystal violet for 30 min. Following staining, the wells were washed three times with 3.5 mL of water to remove unbound crystal violet. After drying, the attached crystal violet was dissolved in 3.5 mL of absolute ethanol, and the optical density (OD) was measured at 600 nm using the ELISA Microplate Reader (Rayto-RT ELISA Microplate Reader). In cases where the absorbance (OD) exceeded 1, the samples were diluted at a 1:10 ratio, and the resulting values were multiplied by 10 to obtain the final measurements. The value obtained from the optical density measurement represented the extent of biofilm formation and is referred to as OD. TSB medium without bacteria served as the negative control. The optical density of the negative control sample was designated as the ODc of the control. For a microorganism to be considered biofilm-positive, its optical density (OD) must exceed the optical density of the control samples (ODc), which serve as the negative control. Also, the *E. coli* strains were classified as non-adherent (OD ≤ ODc), weak (ODc < OD ≤ 2 × ODc), moderate (2 × ODc < OD ≤ 4 × ODc), or strong biofilm-producing (ODc > 4 × ODc), according to their biofilm formation abilities [11,27,28,29]. In addition to OD measurement, biofilm formation was evaluated based on viable cell count in biofilm (see Section 2.4).

### 2.4. Count of Viable Cells in Biofilm of E. coli

Biofilm cells were resuspended in 1 mL of PBS through rigorous pipetting and subsequently serially diluted in PBS. The diluted samples were plated onto tryptic soy agar (TSA; Difco). The plates were incubated at 37 °C for 24–48 h. Following the incubation period, the viable cell counts of *Escherichia coli* biofilms were reported as log CFU/mL [9,28].

### 2.5. Single and Combined Sonication Treatment Against E. coli Biofilm

Cultures grown overnight at 30 °C were diluted to 1% and inoculated into 12-well polystyrene microtiter plates containing 3 mL of tryptic soy broth (TSB). The plates were then incubated at 37 °C for 48 h to allow biofilm formation. Following incubation, the biofilms were washed once with phosphate-buffered saline (PBS) and exposed to 3 mL of treatment solution (PBS, 2% lactic acid, or 2% acetic acid) in an ultrasonic bath (ISOLAB; ultrasonic power: 360 W, frequency: 40 kHz, heating power: 600 W). Treatments were conducted under varying conditions, including 20 °C, 40 °C, and 50 °C, for 2 or 5 min. During the sonication treatment in an ultrasound bath, 12-well polystyrene microplates containing biofilm and inactivation solution were carefully wrapped with stretch film. These microplates were then meticulously placed in the ultrasound bath and subjected to inactivation conditions at 20 °C, 40 °C, and 50 °C for 2 and 5 min, respectively. After sonication and exposure to organic acids, *E. coli* biofilms were washed with PBS. The biofilms remaining at the bottom of the wells after washing were evaluated using optical density measurements and viable cell count enumeration. In the experiments, PBS was a positive control, while pathogen-free TSB was a negative control. This experiment was performed in three technical replicates [9,28,29].

### 2.6. Statistical Analysis and Predictive Modeling

All experiments were conducted in triplicate. Data were analyzed using the SPSS statistical software, version 15.0 (SPSS Inc., Chicago, IL, USA). Results were presented as mean ± standard deviation (SD). Statistical significance (*p* < 0.05) was determined using Duncan’s multiple comparison test following analysis of variance (ANOVA). The relationship between optical density and viable cell count was assessed through simple linear correlation. Furthermore, a multiple linear regression model was developed to predict biofilm inactivation [30].

In a multiple linear regression model, the relationship between two or more independent variables and a dependent variable is explained by fitting a linear equation to observed data. The dependent variable (Y) is related to every independent variable (X). The population regression line for p independent variables X_1_, X_2_, X_3_…, X_P_ is described as μY = β_0_ + β_1_X_1_ + β_2_X_2_ + β_3_X_3_ … + β_p_X_p_. Where μY represents the mean response that varies with the independent variables, the observed values for y fluctuate around their μY and are assumed to have the same standard deviation. The fitted values b_0_, b_1_, b_2_…, bp, predict the parameters β_0_, β_1_, β_2_…, β_p_ of the population regression line. Since the observed values for Y change about their means μY, the multiple regression model involves a term for this variation. This model can be expressed as DATA = FIT + RESIDUAL, where the “FIT” term describes the expression β_0_ + β_1_X_1_ + β_2_X_2_ + β_3_X_3_ … + β_p_X_p_. The “RESIDUAL” term describes the deviations of the observed values y from their means μy, normally distributed with mean 0 and variance. ε represents the notation for the model deviations [31]. Formally, the multiple linear regression model was given in Equation (1):

Y = β_0_ + β_1_X_1_ + β_2_X_2_ + β_3_X_3_ + ε (1)

In the present study, the classical method was used for three independent variables (X_1_ = temperature, X_2_ = time, and X_3_ = solution) and one dependent variable (Y = decrease in cell number or optical density), and the regression equation model given above was utilized (Equation (1)). This regression equation model enabled the estimation of the effects of sonication treatment under varying conditions on the inactivation of *E. coli* biofilms. The accuracy of the models was determined using the parameters R^2^, adjusted R^2^, SSE (the sum of squares error), and RMSE (root mean square error). In general, R^2^ and adj. R^2^ values closer to 1 and RMSE and SSE values closer to 0 indicate a good fit for the data. RMSE and SSE were calculated according to the following formulas (Equation (2) and (3)). Ŷi is predicted values, Yi is observed values, and n is the number of observations [25].

(2) $$\mathrm{RMSE}=\sqrt{\sum_{i=1}^{n}\frac{{\left(Ŷi-Yi\right)}^{2}}{n}}$$

(3) $$\mathrm{SSE}=\sum_{i=1}^{n}{\left(Ŷi-Yi\right)}^{2}$$

## 3. Results

This study utilized data on reducing viable cell count and optical density (OD) to evaluate the efficiency of single and combined sonication treatments in inactivating *E. coli* biofilms. The data established a correlation between OD measurements and viable cell analysis. Additionally, a predictive model for *E. coli* biofilm inactivation was developed based on the results obtained from sonication treatments. The predictive equations estimate the likelihood of *E. coli* biofilm inactivation under varying time and temperature conditions.

### 3.1. E. coli Biofilm Formation on Microplates

Table 1 presents data on *E. coli* biofilm formation on polystyrene microplates. In this study, *E. coli* biofilms exhibited robust formation, with an optical density (OD600) of 1.02. Previous studies have reported optical density values for *E. coli* biofilms in microplates ranging from approximately 0.50 to 2.00 [6,11,17].

### 3.2. The Effect of Single Sonication and Combined Sonication Treatment Against E. coli Biofilms

In this study, polystyrene plates containing *E. coli* biofilm were subjected to a single sonication treatment with PBS and a combined sonication treatment with lactic acid or acetic acid at 20, 40, and 50 °C for 2 and 5 min. Subsequently, biofilm inactivation was assessed based on viable cell counts and the optical density (OD) of the remaining *E. coli* biofilms on the plates. The viable cell count analysis results aligned with the OD analysis, showing a similar reduction trend in *E. coli* biofilm inactivation (Table 2 and Table 3). Treatment temperature, treatment time, and the type of washing solution (lactic acid, acetic acid, or PBS) significantly influenced *E. coli* biofilm destruction (*p* < 0.05). A stronger anti-biofilm effect was observed with increased treatment time and temperature. The combined sonication treatment with organic acids also resulted in greater biofilm reduction. Lactic or acetic acid contributed to an additional 1.5 to 3 log CFU/mL reduction in *E. coli* biofilm on polystyrene surfaces compared to sonication alone.

Table 2 presents the reduction in *E. coli* biofilm, measured as viable cell counts, following sonication treatment under various conditions. The reduction ranged from 0.43 to 6.21 log CFU/mL. The lowest reduction was observed with single sonication at 20 °C for 2 min, while the highest reduction occurred with combined thermosonication treatment at 50 °C for 5 min. Organic acid treatments were statistically more effective than PBS treatments in detaching *E. coli* biofilms (*p* < 0.05). However, the type of organic acid used (lactic acid or acetic acid) did not significantly influence biofilm reduction (*p* > 0.05), indicating that either organic acid can effectively remove *E. coli* biofilm.

Table 3 presents *E. coli* biofilm detachment, measured in terms of optical density (OD), after sonication under various conditions. Treatments incorporating organic acids, prolonged exposure times, and higher temperatures resulted in greater *E. coli* biofilm detachment on polystyrene plates (*p* < 0.05). The most significant biofilm removal, a decrease of 0.72 OD, was observed with the combination of sonication and lactic acid for 5 min at 50 °C. In the present study, combining lactic acid and acetic acid with sonication at 20 °C for 5 min resulted in greater biofilm detachment (0.45 to 0.60 OD). These findings, consistent with viable cell count analyses, confirmed that combined treatments of organic acids and sonication were more effective for biofilm removal than treatments with organic acids alone.

### 3.3. Relationship Between Biofilm Inactivation Tests

**Figure 1:**
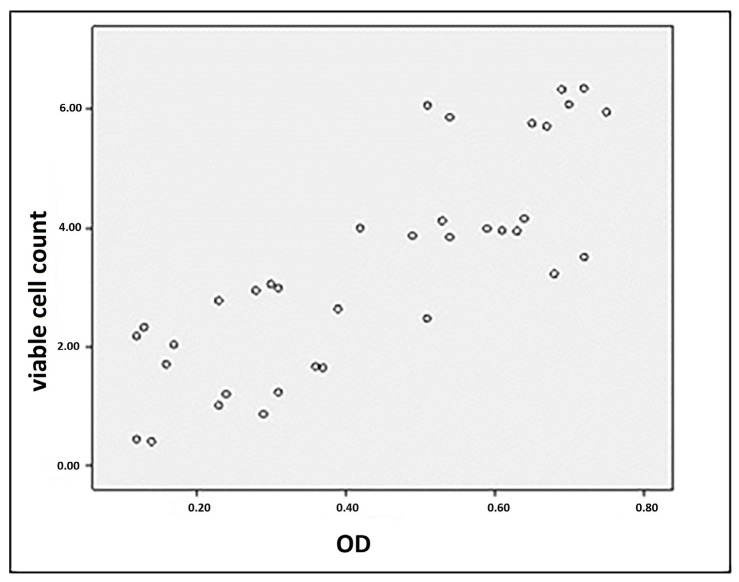
Relationship between biofilm inactivation tests based on viable cell count and OD.

To obtain more precise insights into cell reduction, the present study evaluated biofilm inactivation using viable cell counts in addition to OD. Furthermore, the relationship between biofilm inactivation tests was assessed using simple linear correlation analysis. A scatter plot was generated to examine the relationship between *E. coli* biofilm inactivation tests. OD values were plotted on the *x*-axis and viable cell counts on the *y*-axis (Figure 1). The results revealed a significant positive linear correlation (r = 0.817, *p* < 0.01) between viable cell counts and OD measurements. The plate counting and OD methods were compared to assess microbial growth. The comparison demonstrated that OD can be an alternative technique for estimating viable cell counts. Microbial growth rates can be inferred from the slopes of the OD profiles. These findings suggest that both inactivation test methods—viable cell counts and OD—help compare sonication treatments. However, analyses based on viable cell counts are recommended, as they provide more accurate inactivation data.

### 3.4. Modeling E. coli Biofilm Elimination with Regression Analysis

The present study developed prediction models for biofilm inactivation, measured in terms of viable cell count and optical density (OD), using multiple linear regression analyses. The classical method was employed with three independent variables—temperature (X_1_), time (X_2_), and solution (X_3_)—and one dependent variable, representing the decrease in cell number or OD (Y). The following regression equation model was applied to estimate the effects of sonication treatment under varying conditions on *E. coli* biofilm inactivation. These models demonstrated the capability to predict the resistance of *E. coli* biofilms to sonication treatment across different conditions.

The regression equation model (Equation (4)), which describes the biofilm inactivation rate (%) based on viable cell count, is presented below. Temperature, time, and solution significantly correlated with the biofilm inactivation rate (R = 0.921, R^2^ = 0.847, Adjusted R^2^ = 0.833). Collectively, these independent variables explained 84% of the variability in the biofilm inactivation rate. The decontamination efficiency of each variable displayed varying levels of statistical significance. Standardized regression coefficients (β) revealed the relative importance of the independent variables: solution (β = 0.569), time (β = 0.555), and temperature (β = 0.464). Significance tests for the regression coefficients indicated that all independent variables (temperature, time, and solution) had a statistically significant effect (*p* < 0.01) on the inactivation rate. Additionally, data from viable cell counts indicated that *E. coli* biofilms were more sensitive to organic acids than other factors.

Y = −44.679 + 0.769X_1_ + 7.642X_2_ + 14.400X_3_ (4)

The regression equation model (Equation (5)), describing the biofilm removal rate (%) as a function of optical density, is presented below. The variables temperature, time, and solution demonstrated a significant relationship with the biofilm removal rate (R = 0.898, R^2^ = 0.806, Adj. R^2^ = 0.800). Together, these independent variables account for 80% of the variance in the inactivation rate. Standardized regression coefficients indicate the relative importance of the independent variables, ranked as time (β = 0.647), temperature (β = 0.601), and solution (β = 0.163). Significance tests of the regression coefficients revealed that temperature and time significantly affected the biofilm removal rate at the 99% confidence level (*p* < 0.01). In comparison, the solution variable was significant at the 95% confidence level (*p* < 0.05). Furthermore, optical density (OD) measurements indicated that *E. coli* biofilms were more sensitive to treatment time than the other variables.

Y = −30.478 + 0.961X_1_ + 8.603X_2_ + 3.970X_3_ (5)

In the present study, the prioritization of independent variables (temperature, time, and solution) varied depending on the test methods employed, including viable cell count and optical density (OD) measurement (Equations (4) and (5)).

**Figure 2:**
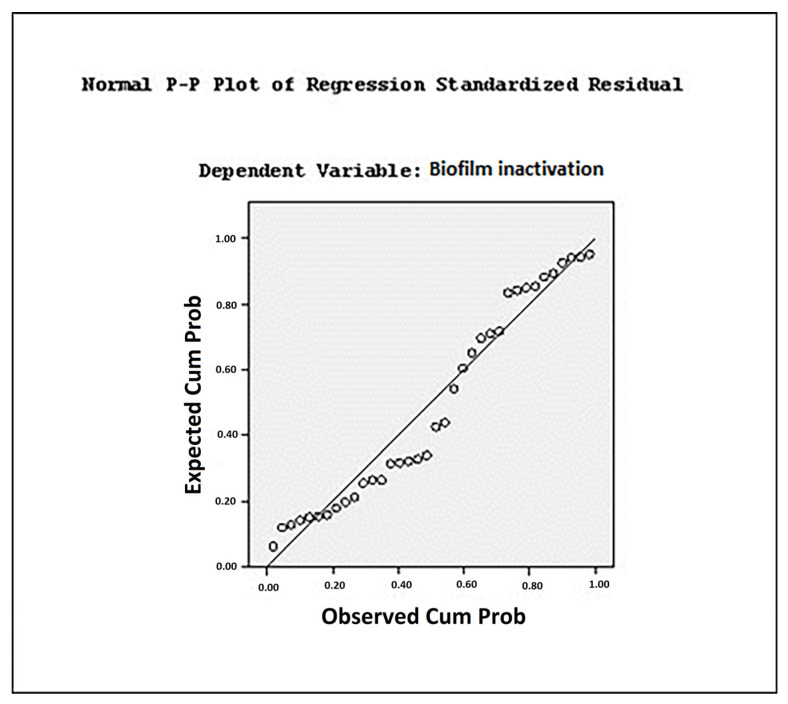
Scatter plot of observed and predicted values in terms of viable cell counts.

**Figure 3:**
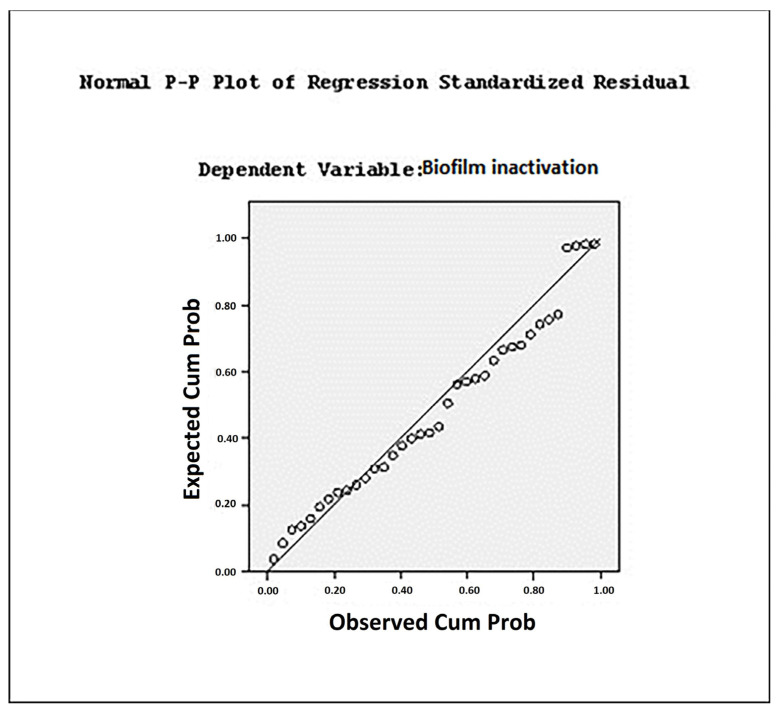
Scatter plot of observed and predicted values in terms of OD.

Scatter plots of observed versus predicted values, shown in Figure 2 and Figure 3, were used to evaluate the suitability of the predictive models. These plots depict the probability of *E. coli* biofilm inactivation following single and combined sonication treatments derived from the predictive equations (Equations (4) and (5)). In all models, the *p*-value was <0.05 or <0.01, and the multiple correlation coefficients (R^2^) were 0.84 and 0.80, indicating a strong correlation between predicted and observed values. The fitting curves of prediction models, expressed in terms of viable cell count and optical density (OD), showed similar reduction trends, as illustrated in Figure 2 and Figure 3. The data indicate that the prediction model based on viable cell count provided a better fit for describing biofilm elimination under each treatment, as evidenced by the higher R^2^ value (0.84).

## 4. Discussion

### 4.1. E. coli Biofilm Formation on Microplates

Surface materials support the growth of biofilm in the following order: latex > polyethylene > PVC > polypropylene > stainless steel > glass. The glass surface was free of crevices, while the stainless steel surface exhibited numerous oblique, straight-line, narrow crevices. The plastic surface displayed convex patterns, with a higher concentration of *E. coli* O157:H7 observed at the edges of these patterns. The wood surface contained many pores and deep crevices, which supported the development of dense *E. coli* O157:H7 communities [32]. Polystyrene and stainless steel are commonly used as food contact surfaces in the food industry and households. However, biofilm formation by *S. aureus* was reported to be higher in polystyrene (65.7%) than in stainless steel (63.1%). At 37 °C, *S. aureus* was classified as a moderate biofilm producer on polystyrene and a weak biofilm producer on stainless steel [5]. The effect of surface materials, such as polystyrene microtitration plates and stainless steel, on *E. coli* biofilm elimination with organic acid treatments was evaluated by Akbaş and Çağ [15]. *E. coli* biofilm elimination reportedly improved on stainless steel than polystyrene surfaces. Conversely, Avila et al. [3] used stainless steel and polystyrene surfaces as food contact materials for biofilm formation assays and found no significant differences in microbial counts between the surfaces. The present study investigated biofilm detachment on polystyrene surfaces, whereas most of the existing literature focuses on biofilm removal from stainless steel. Limited research has specifically addressed biofilm detachment from plastic materials, particularly polystyrene [32]. Polystyrene, known for its high hydrophobicity, is widely utilized as a food contact surface in the food industry, including applications such as conveyor belts, cutting boards, gaskets, packaging, belts, pipes, processors, and containers. While generally considered safe under normal usage conditions, polystyrene may release harmful substances when subjected to elevated temperatures or extreme pH environments, such as acidic or alkaline conditions [33,34].

Consistent with the present findings, earlier research documented biofilm populations on various surfaces, including stainless steel, glass, plastic (polyethylene), and wood, at 8.5, 8.8, 8.7, and 9.6 log CFU/coupon, respectively [1]. The ability of *E. coli* to form biofilms in microplates can vary depending on factors such as live cell concentration, microbial growth phase, microplate properties (e.g., well number and size, material type), growth medium, and incubation conditions [15]. For instance, Nesse et al. [4] observed that *E. coli* can form biofilms on various food processing surfaces, including stainless steel, glass, and polystyrene, with stainless steel and polystyrene promoting higher levels of biofilm formation than glass.

### 4.2. The Effect of Single Sonication and Combined Sonication Treatment Against E. coli Biofilms

A simple decontamination procedure using antimicrobial substances proved insufficient for inactivating highly resistant biofilms. Consequently, some studies have incorporated sonication alongside these compounds to enhance bactericidal effectiveness. However, prolonged exposure to sonication or antimicrobial substances may cause mechanical damage to food contact surfaces [35]. For instance, using a single organic acid treatment requires a specific concentration, which often results in higher costs, undesirable off-flavors in the product, nutrient loss, or damage to food contact surfaces with minor cracks and scratches. Consequently, combining organic acid treatment with other inactivation techniques can enhance microbial inactivation while reducing the required concentration of organic acids [16,21,35]. Thermosonication, a combined treatment of heat and ultrasound, offers more efficient sanitation within a shorter time frame and causes minimal damage to food contact surfaces compared to power ultrasound [35,36,37]. Sonication effectively disrupts the polysaccharide and protein components of bacterial biofilms, altering the protein composition of the detached extracellular polymeric substances (EPSs). The polysaccharides in the loosely bound EPSs typically serve as the first barrier protecting microbial cells from the detrimental effects of chemical disinfectants. In the absence of this extracellular polysaccharide protection, microbial cells within biofilms become more vulnerable to disinfectants [12].

These findings align with previous studies, which reported greater microbial susceptibility to combined sonication treatments than single sonication treatments [2,35,37,38]. The results suggest that a similar decontamination rate can be achieved with reduced treatment time by combining thermosonication with organic acids.

Consistent with the present study, Yuk et al. [39] and Stopforth et al. [13] reported no significant difference in biofilm destruction based on the type of organic acid. In contrast, Ji et al. [16] found that lactic acid significantly reduced mature *E. coli* biofilms compared to acetic acid. According to Park and Chen [6], the serotypes of *E. coli* strains influence biofilm resistance to antimicrobial substances. Their study investigated the treatment of biofilms formed by various *E. coli* serotypes on polystyrene surfaces with 2% lactic acid and 2% acetic acid. The optical density (OD) reduction in *E. coli* biofilms ranged from 0.1 to 0.3 OD, depending on the strain’s serotype. The study found the type of organic acid to have an insignificant antibiofilm effect, consistent with the results of our research.

Thermosonication at mild temperatures and using organic acids at low concentrations is suitable for decontaminating food contact surfaces. However, despite its advantages, using thermosonication and organic acids also presents certain disadvantages for disinfection. High temperatures and elevated concentrations of organic acids can damage surface materials or equipment used in the food industry. Therefore, the concentration of antimicrobial substances, treatment duration, and treatment temperature must be carefully controlled to avoid harming food contact surfaces [16,21]. The mechanism of microbial inactivation with sonication is based on the damage to the cell wall, notably the biofilm matrix and cytoplasmic membrane [25]. Sonication treatment partially affected the outer layer of the biofilm matrix, while cells in the inner layer were protected from the sanitizer, allowing them to survive [12]. The antibiofilm mechanism of organic acids is linked to their undissociated form and pH. Undissociated organic acid molecules damage microbial cell membranes, leading to microbial inhibition. The dissociation of organic acids varies depending on the duration and temperature of ultrasonication. For instance, dissociation increases with rising temperatures and prolonged sonication [40]. In this study, ultrasonication was performed at a maximum of 50 °C for 5 min to minimize the dissociation of organic acids, as higher temperatures and longer treatment times may accelerate this process. Limiting treatment time and temperature is crucial to preventing damage to food contact surfaces, including the formation of tiny cracks and scratches. Polymers such as polyethylene and polystyrene, often used in the food industry, may degrade because of stress factors including heat, light, chemicals, or any other applied force. Degradation causes a reduction in the molecular weight of the polymer. Decontamination treatments have potential to introduce detrimental changes such as scratches to the polystyrene surface as a result of the degradation of polymers. The ultrasonic and thermal degradation process of polystyrene depends on the ultrasonic amplitude, temperature, dissolved gasses, and the chemical solution used. The degradation of polystrene and polyethylene generally requires treatment at high temperatures between 350–450 °C [41]. For this reason, thermosonication under mild conditions including the use of low frequency, low temperature, low time, and disinfectants at low concentrations was suggested for the decontamination of food contact surfaces [16,21,41,42]. Additionally, combined treatment is preferable since this prevents the damaging of surface materials due to extremely intense treatment conditions (longer processing time, higher processing temperature, and higher amount of antimicrobial agents) [22].

The efficacy of decontamination techniques depends on several factors, including microbial load, sonication duration, treatment temperature, intensity, and frequency [25]. For example, Lee et al. [19] demonstrated that a 5-log reduction in *E. coli* K12 was achieved with a single thermosonication treatment at 61 °C for 4 min. In contrast, the same level of *E. coli* reduction was accomplished in a significantly shorter time (0.075 min) when thermosonication was combined with pressure treatment. Similarly, Kwak et al. [36] reported a 0.97 log CFU/g reduction in *E. coli* O157:H7 using thermoultrasound and 2% calcium propionate at 50 °C for 10 min. Turhan and Polat [43] also observed enhanced *E. coli* biofilm destruction through the synergistic effects of sonication combined with organic acids, including lactic, acetic, malic, and citric acids. In another study, single ultrasound treatment (500 kHz) and ultrasound treatment combined with nisin (500 kHz) resulted in approximately 1- and 2-log reductions in *E. coli* within 20 min, respectively [44]. Fan et al. [35] found that thermosonication pretreatment (15 min at 55 °C) enhanced the sporicidal activity of UV irradiation in suspension, achieving additional reductions of 2.74 to 3.78 logs. Consistent with these findings, the present study confirmed that combining thermosonication with other inactivation methods was more effective for microbial reduction.

Park and Chen [6] also studied *E. coli* biofilm on polystyrene plates treated with lactic acid and acetic acid for 20 min, reporting biofilm removal ranging from 0.01 to 0.26 OD and 0.03 to 0.21 OD, respectively.

The antibacterial mechanism of organic acids, such as acetic and lactic acids, involves lowering the pH of the environment. This activity is primarily attributed to the dissociated and non-dissociated forms of organic acids, with the uncharged, non-dissociated forms primarily responsible for their antibacterial effects. According to the “weak organic acid theory”, these acids disrupt bacterial membrane stability by reducing membrane-associated molecular interactions, leading to pore formation and rapid cell death. Specifically, lactic acid destabilizes the membrane, disrupts the transmembrane proton motive force, denatures acid-sensitive proteins and DNA, and interferes with metabolic and anabolic processes [45]. Additionally, combining organic acids and sonication reduces *E. coli* resistance to sonication by creating a lower pH environment [46]. Previous studies have corroborated that *E. coli* sensitivity to sonication increases with longer treatment times and higher temperatures [19,33,47,48].

### 4.3. Relationship Between Biofilm Inactivation Tests

In previous studies, optical density (OD) measurement has frequently been used to evaluate microbial inactivation efficiency [15,43]. OD analysis is faster than plate counting; however, based on turbidity, it accounts for all bacterial biomass, including dead and live cells [49,50]. Consequently, viable cell analyses considering only live cells yield more accurate and reliable results [51]. For example, a previous study reported 68–86% *E. coli* biofilm inactivation based on viable cell counts and 52–60% *E. coli* biofilm removal based on OD after treatment with 2% organic acids (malic acid, citric acid, and gallic acid) for 5, 10, and 20 min. These findings confirmed that the OD method measures all bacterial cells regardless of viability [15].

According to present data, analyses based on viable cell counts are recommended, as they provide more accurate inactivation data. Similarly to those obtained in the present study, Loske et al. [49] stated that a correlation (R: 0.95) between the data from the plate count method and turbidimetric analysis was obtained.

### 4.4. Modeling E. coli Biofilm Elimination with Regression Analysis

Predictive modeling of pathogenic bacteria during decontamination provides valuable insights for quantitatively assessing microbial risks. It also suggests tools for comparing the efficacy of different inactivation methods (e.g., [24]). Although predicting the inactivation of foodborne pathogens with complete accuracy using mathematical models is impossible, these models serve as valuable tools. They support process optimization to enhance food safety (e.g., [32,52]).

The determination coefficient (R^2^) and adjusted determination coefficient (Adj.R^2^) were utilized to judge how much the variability of the response variable can be affected by the independent variables. R^2^ measures the proportion of variance explained by independent variables while Adj.R^2^ penalizes addition of irrelevant variables, preventing overfitting. Adj.R^2^ ensures a more accurate measure for models with multiple predictors. It is always lower than R^2^. Generally, R^2^ and Adj.R^2^ values close to 1 indicate a strong predictive ability of the model [14,30]. Conversely, low Adjusted R^2^ (Adj. R^2^) values, particularly those below 0.7, suggest the model’s inadequacy to account for the effects of the independent variables on the response [24]. For instance, Kavuncuoğlu et al. [31] reported that R^2^ values below 0.8 indicate poor agreement between predicted and experimental data. The regression model in this study achieved an R^2^ of 0.847 and an Adj. R^2^ of 0.833, demonstrating a strong fit of the regression equation to the data. Thus, the model effectively predicted the impact of sonication time, temperature, and treatment solution on *E. coli* biofilm inactivation.

Carvalho et al. [14] investigated the antibiofilm activity of malic acid and citric acid on *E. coli* adhesion to polystyrene surfaces at varying temperatures (4, 12, and 25 °C), compound concentrations, and contact durations (5 and 10 min). Both malic acid and citric acid demonstrated antibiofilm activity under all tested conditions, with temperature having a more significant impact than contact time. The efficacy of microbial inactivation methods depends on various factors, including the type of treatment, the physiology and type of the target microorganism, surface characteristics, treatment duration, temperature, pH, and concentration of agents. In the food industry, surface damage and disinfectant residues are undesirable. Combined inactivation techniques have been applied to address the limitations of single inactivation techniques, such as prolonged treatment time, elevated temperature, and high disinfectant concentrations [53].

Nevertheless, the numerous factors influencing biofilm inactivation necessitate mathematical models. Predictive modeling, mainly through sonication, has been identified as an effective strategy to explain microbial inactivation, as noted in previous research [26,38]. Prior studies demonstrated good statistical alignment between models and experimental results for microbial inactivation via sonication. However, to our knowledge, no research has modeled eliminating *E. coli* biofilm using combined thermosonication and organic acids through a multiple regression approach. Regression models have been employed in various studies investigating *E. coli* biofilm inactivation on polystyrene and other food-contact surfaces, utilizing disinfectants such as essential oils, chemical antimicrobial substances, ultrasound, and thermal inactivation techniques [2,32]. However, predictive modeling of biofilm inactivation using thermosonication treatment remains limited.

Newly developed models tailored to specific inactivation methods better fit experimental and predicted data [25,31,54]. For instance, a previous study utilized linear and non-linear regression models to predict *E. coli* inactivation, suggesting that a multiple linear regression model (R^2^ = 0.86) offered superior predictive accuracy [52]. Similarly, the present study’s prediction of *E. coli* biofilm inactivation demonstrated high accuracy with a multiple regression model. The R^2^ values for the fitted models, based on viable cell count and optical density (OD), were 0.84 and 0.80, respectively. This indicates that prediction modeling based on viable cell count was more accurate than OD-based. The findings support the hypothesis that regression modeling using live cell counts is more reliable than OD measurements [49,50].

In multiple linear regression analyses, residual values are examined to assess the variability unexplained by the model. The residual represents the difference between the observed value and the predicted value, specifically the deviation between the observed value and the value on the regression line. The residual sum of squares measures the extent to which the regression line fits the data. A smaller residual sum of squares indicates a better fit [55]. The residual value represents the deviation between observed values and the predicted values of the dependent variable. A normal P–P plot was employed to assess the normality of residual values. Data point closer to the diagonal line in the plot indicate that the residual values closely follow a normal distribution. Conversely, deviations from the diagonal line suggest that the data do not conform to normality, highlighting the extent of deviation from the normal distribution [30].

## 5. Conclusions

The present study demonstrated that the combined treatment of sonication with organic acids significantly enhanced the removal of *E. coli* biofilm from polystyrene surfaces compared to a single thermosonication treatment. Under varying sonication conditions, the number of viable *E. coli* biofilm cells decreased by 0.43–6.21 log CFU/mL, while the optical density at 600 nm (OD600) decreased by 0.13–0.72. A significant positive correlation was observed between viable cell counts and optical density measurements. Combining organic acids and thermosonication at 50 °C for 5 min was identified as the most effective method for reducing *E. coli* biofilm. However, none of the treatments completely removed *E. coli* biofilms from polystyrene surfaces. This finding is critical for improving the efficacy of *E. coli* biofilm inactivation. Regression modeling of biofilm elimination provided accurate estimates of microbial reduction, with scatter plots of viable cell count data showing a closer relationship between observed and predicted values compared to OD600 data. The prediction models have potential applications in risk assessment and selecting appropriate sonication treatments in the food industry. Developing predictive models for *E. coli* biofilm inactivation on food contact surfaces under various sonication conditions contributes to risk assessment throughout the food chain, from farm to fork. Accurate models enable precise predictions, broadening their practical applications. Future research should focus on the inactivation of biofilms formed by foodborne pathogenic bacteria on various food products using thermosonication under different conditions, such as varying antimicrobial substances, temperatures, and exposure times. Additionally, non-linear regression models should be applied to achieve more precise predictions of biofilm inactivation and compared with other predictive models.

## Figures and Tables

**Table 1:** *E. coli* biofilm formation on polystyrene microplates.

	OD	Viable Cell Counts (log CFU/mL)	Biofilm Formation
Biofilm	1.02 ± 0.03	8.56 ± 0.08	+
Negative control	0.14 ± 0.00	0.00 ± 0.00	-

**Table 2:** Reduction in viable cells of *E. coli* biofilm with combined and single ultrasonication treatment (log CFU/mL).

Sonication Conditions	Control (PBS)	Lactic Acid (2%)	Acetic Acid (2%)
20 °C–2 min	0.43 ± 0.03 ^eB^	2.18 ± 0.20 ^eA^	1.95 ± 0.34 ^dA^
40 °C–2 min	1.12 ± 0.13 ^dB^	2.88 ± 0.15 ^dA^	3.01 ± 0.08 ^cA^
50 °C–2 min	1.66 ± 0.01 ^cB^	4.06 ± 0.14 ^cA^	3.99 ± 0.20 ^bA^
20 °C–5 min	1.05 ± 0.26 ^dB^	3.98 ± 0.02 ^cA^	3.94 ± 0.09 ^bA^
40 °C–5 min	2.56 ± 0.11 ^bB^	5.74 ± 0.04 ^bA^	5.96 ± 0.15 ^aA^
50 °C–5 min	3.37 ± 0.19 ^aB^	6.21 ± 0.19 ^aA^	6.14 ± 0.26 ^aA^

A–B: the difference between the values indicated with different letters in the same row is statistically significant (*p* < 0.05). a–e: the difference between the values indicated with different letters in the same column is statistically significant (*p* < 0.05).

**Table 3:** Reduction in optical density of *E. coli* biofilm with combined and single ultrasonication treatment (OD).

Sonication Conditions	Control (PBS)	Lactic Acid (2%)	Acetic Acid (2%)
20 °C–2 min	0.13 ± 0.02 ^eA^	0.15 ± 0.03 ^dA^	0.14 ± 0.03 ^eA^
40 °C–2 min	0.23 ± 0.00 ^dA^	0.27 ± 0.06 ^cA^	0.29 ± 0.01 ^dA^
50 °C–2 min	0.37 ± 0.01 ^cbC^	0.63 ± 0.01 ^bA^	0.54 ± 0.01 ^bB^
20 °C–5 min	0.30 ± 0.01 ^cdC^	0.60 ± 0.01 ^bA^	0.45 ± 0.05 ^cB^
40 °C–5 min	0.45 ± 0.09 ^bB^	0.66 ± 0.02 ^abA^	0.52 ± 0.02 ^bcAB^
50 °C–5 min	0.70 ± 0.03 ^aA^	0.71 ± 0.01 ^aA^	0.72 ± 0.04 ^aA^

A–C: the difference between the values indicated with different letters in the same row is statistically significant (*p* < 0.05). a–e: the difference between the values indicated with different letters in the same column is statistically significant (*p* < 0.05).

## Data Availability

The datasets presented in this article are not readily available because the data are part of an ongoing study.
